# Direct interaction of food derived colloidal micro/nano-particles with oral macrophages

**DOI:** 10.1038/s41538-017-0003-3

**Published:** 2017-10-30

**Authors:** Lijing Ke, Huiqin Wang, Guanzhen Gao, Pingfan Rao, Lei He, Jianwu Zhou

**Affiliations:** Food Nutrition Sciences Centre, Zhejiang Gongshang University, Hangzhou, China

**Keywords:** Mucosal immunology, Nanoparticles

## Abstract

Like any typical food system, bone soup (or broth), a traditional nourishing food in many cultures, contains a colloid dispersion of self-assembled micro/nano-particles. Food ingestion results in the direct contact of food colloidal MNPs with immune cells. Will they ever interact with each other? To answer the question, MNPs and NPs were separated from porcine bone soup and labeled with Nile Red, and their uptake by murine oral macrophages and its consequent effects were investigated. Colloidal particle samples of UF-MNPs and SEC-NP were prepared from porcine bone soup by ultrafiltration (UF) and size-exclusion chromatography, respectively. Their mean hydrodynamic diameters were 248 ± 10 nm and 170 ± 1 nm with dominant composition of protein and lipid. Particles in both samples were found to be internalized by oral macrophages upon co-incubation at particle/cell ratios of 14,000/1. In normal oral macrophages, the particle uptake exerted influence neither on the cellular cytosolic membrane potential (*V*_mem_) nor mitochondrial superoxide level, as were indicated with fluorescent dyes of DiBAC_4_(3) and MitoSOX Red, respectively. However, when oral macrophages were challenged by peroxyl radical inducer AAPH, the engulfment of UF-MNPs and SEC-NPs mitigated the peroxyl radical induced membrane hyperpolarization effect by up to 70%, and the suppression on the oxygen respiration in mitochondria by up to 100%. Those results provide evidence of the direct interaction between food colloidal particles with immune cells, implying a possible new mode of food-body interaction.

## Food ingestion: Colloidal particles derived from bone soup interact with immune cells

The uptake of food colloidal particles by oral macrophages can mitigate the peroxyl radical induced membrane hyperpolarization effect and suppress the oxygen respiration in mitochondria. Jianwu Zhou and coworkers from Zhejiang Gongshang University, China, separated micro/nano particles of 248 ± 10 nm and 170 ± 1 nm in diameter from porcine bone soup via membrane ultrafiltration and gel-filtration, respectively. By labeling the colloidal particles with hydrophobic fluorescent dyes, they tracked their internalization by oral macrophages upon co-incubation. It was found the food colloidal particles ingestion had no influence on the cell plasma membrane potential and mitochondrial superoxide level of the normal oral macrophages. However, when macrophages were irritated by peroxyl radical inducer 2,2′-azobis (2-amidinopropane) dihydrochloride, the food micro/nano particles exhibited a pacifying effect. This finding reveal a possible new mode of food-body interaction.

## Introduction

As a multiphase dispersion system, foods generally have high content of amphiphilic compounds, among which the covalent and non-covalent interaction occurs, influenced by various bonding forces in the food processing, e.g., heating, extracting, and homogenization. Unexpected but reasonably, the molecular interaction produces a massive amount of self-assembled colloidal particles, whose size distribution ranged from micrometers to nanometers, namely micro/nano-particles (MNPs)^1^. Meanwhile, food compositions are promising building materials for preparation of organic nano-assemblies including MNPs, due to their surprisingly good biocompatibility, functionality, stability, and less toxicity as compared to the chemically synthetic micro/nano-materials. For instance, the organic nanoparticles from green tea extracts are excellent nanocarrier of chemotherapeutic agents for anticancer therapy;^2^ milk protein nanoparticles for the improved oral delivery of folic acid^3^ and ω-3 polyunsaturated fatty acids;^4^ gelatin-based nanoparticles retained the biological activity of encapsulated epigallocatechin gallate (EGCG);^5^ self-assembled nanoparticles (NPs) of the licorice protein reduce the toxicity of aconitine by encapsulation.^6^

Moreover, the self-assembled MNPs and NPs have been found in daily food, whether cooked in kitchen or processed in factory, but little has been known about their characteristic and functions. The pioneering studies on this direction include the isolation and identification of ephedrine loaded NPs from a Chinese herbal extract were;^7^ the formation of unifying MNPs during the gentle and slow cooking process of porcine bone soup.^8^

The mucosa of alimentary tract is the interface where human body is having direct contact with food and drink, and where many vital immune responses are initiated. Mucosa-associated lymphoid tissues (MALT) is the essential compartment of the alimentary tract, accounting for two major functions: acts as immune defense to protect alimentary tract from pathogens and other external stimuli, and participates in absorption and transportation of food and chylomicrons. It has been widely known to be involved in uptake of exogenous particulate matter, including microparticles in food,^9^ polymerized liposomes^10^ and self-assembled NPs of cell penetrating peptide for insulin oral delivery.^1^ Moreover, NPs can modulate inflammation on the site of mucosa without being translocated into blood circulation, as is demonstrated in the orally ingested redox (MeO-PEG-b-PMOT) NPs which accumulate in colonic mucosa, scavenge ROS and therefore reduce colitis in mice.^11^ The uptake of particles is mediated by cells like enterocytes, microfold cells, or macrophages.^12^

However, the direct interaction between self-assembled colloidal MNPs in real food and alimentary tract mucosa remained largely unknown. We have hypothesized that food antioxidant nanoparticles can directly interact with MALT, including macrophages and therefore be of great physiological importance.^13^ Playing key role in mediating innate or adaptive immune responses, macrophage is of particular interest for studying the interaction between micro/nano-materials and MALT. Macrophages capture and translocate organic or nonorganic particles, e.g., lipid liposome,^14^ and TiO_2_ microparticles or nano-particles,^15^ polystyrene particles with various biomacromolecule surface coating,^16^ and glyco-NPs in different shapes.^17^ Interaction between food-derived MNPs with macrophages is therefore a very tempting topic for a better understanding of lymphatic uptake of food compositions.

Bone soup and/or broth is worldwide appreciated for its multi-purpose nourishing functions, besides the joyful taste, particularly in facilitating prevention and treatment of inflammatory bowel disease, immune malfunction,^18,19^ glucose intolerance, and insulin insensitivity in vivo.^20^ Porcine bone soup exhibits intracellular antioxidant activities in epithelial cells, e.g., colon epithelial cell Caco-2 and Madin–Darby Canine Kidney (MDCK) cells, challenged by AAPH-induce peroxyl radicals.^21^ Despite its obviously low content of the known nutrients, bone soup MNPs are created during cooking, possibly rendering the soup with new functions not available in the native constituents.

To testify our hypothesis, here we set off to explore the uptake of porcine bone soup MNPs and NPs by murine (Wistar rats) macrophages from oral cavity, and the consequent influences on the cellular redox status indicated by plasma membrane potential and mitochondrial reactive oxygen species (mito-ROS). For tracking and observing the uptake of UF-MNPs and SEC-NPs, the particles were stained with hydrophobic fluorescent probe, i.e., Nile red (labeling the constitutive lipids), prior to being introduced to macrophages in vitro. The cell plasma membrane potential (*V*_mem_) and mitochondrial superoxide level of oral macrophages were accessed with fluorescent dyes DiBAC_4_(3) and MitoSOX Red (also named Mito-HE), respectively, at either presence or absence of bone soup MNPs and peroxyl radical inducer AAPH. The variation of fluorescence was quantified simultaneously by a fluorometric imaging plate reader.

Investigation on the direct interaction of porcine bone soup MNPs and NPs with macrophages will not only elucidate its influence on immunity but more importantly reveal a possible new mode of food immune modulation.

## Results

### Preparation and characterization of bone soup UF-MNPs and SEC-NPs

**Fig. 1:**
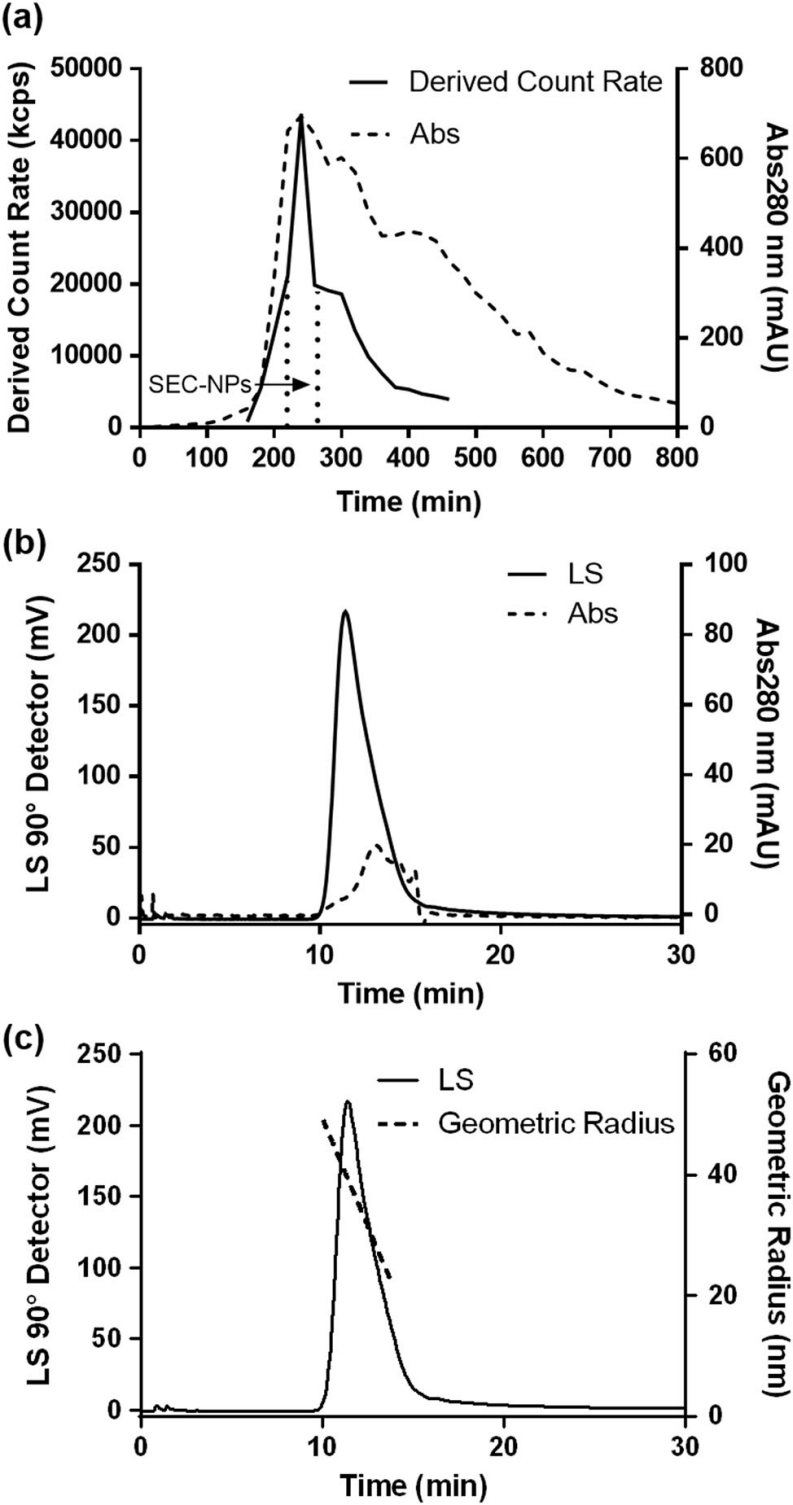
SEC chromatographic isolation of SEC-NPs from porcine bone soup. Bone soup was fractioned by a Sephacryl S-1000 SF column (1.0 × 100 cm) equilibrated with 0.02 M phosphate buffer (pH 7.4). The column was eluted with the same buffer at a flow rate of 0.50 mL/min at 40 °C with UV and Zetasizer Nano-ZS detector (Malven, UK). The purity and molar mass of SEC-NPs was determined by a SEC-MALLS (Size-Exclusion HPLC coupled with Multi-Angle Laser Light Scattering). A TSK gel G6000PWxl HPLC column (0.78 × 300 cm, Tosoh Bioscience, Japan) was equilibrated and eluted with phosphate buffer (0.1 M, pH7.4) at flow rate 0.80 mL/min. **a**: SEC chromatogram (Sephacryl S-1000 SF column) of the porcine bone soup is presented as the Derived Count Rate (solid line) and UV absorptance at 280 nm (dash line). The fraction of NPs was collected and named SEC-NPs, as indicated by two dot line. **b**: The SEC-MALLS chromatogram (TSK gel G6000PWxl HPLC column) of SEC-NPs fraction is presented as the light scattering intensity at 90° of MALLS (solid line) and UV absorptance at 280 nm (dash line). **c**: The solid line is the SEC-MALLS chromatogram wherein the dash line represents the geometric radius distributions (22–49 nm) of the light scattering peak

**Fig. 2:**
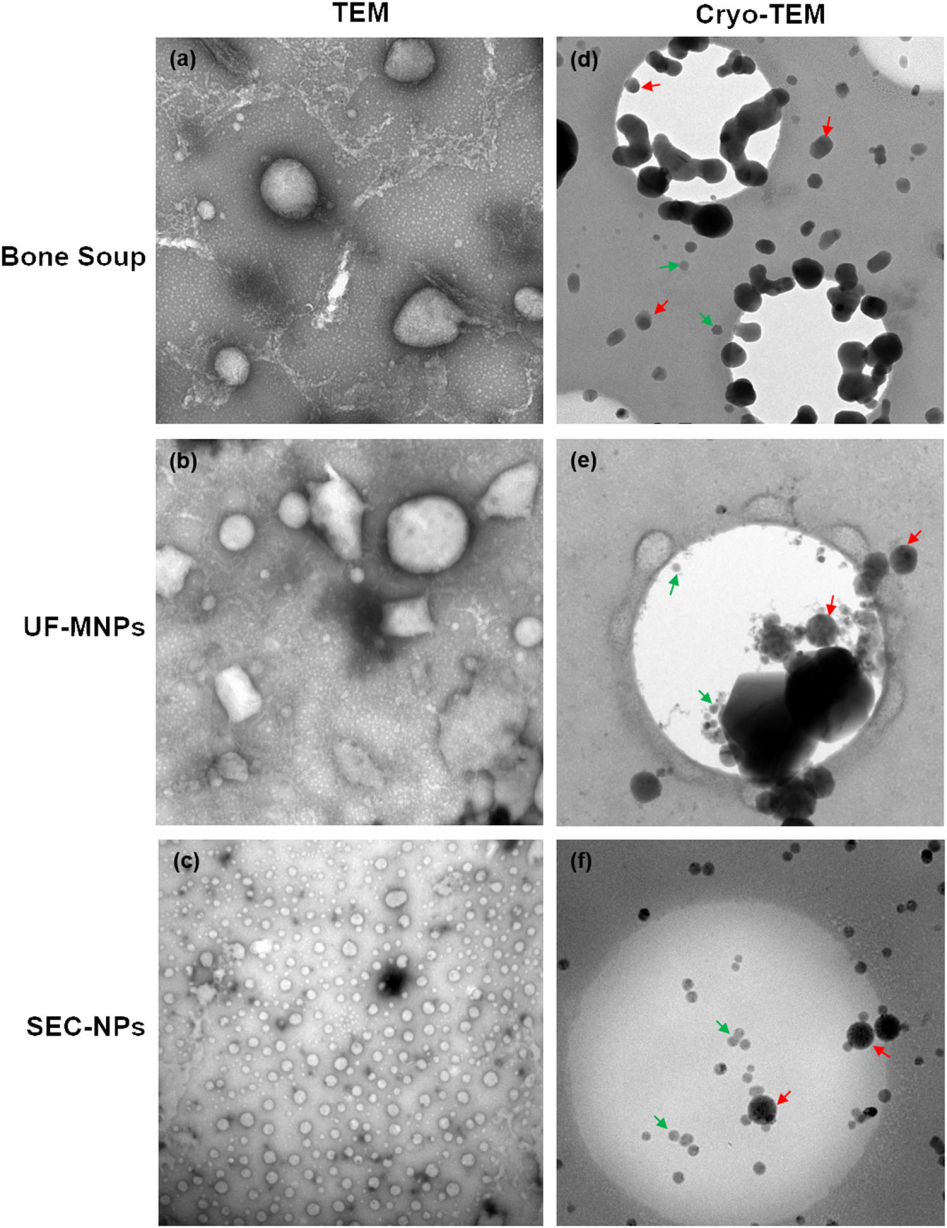
TEM and Cryo-TEM micrographs of food derived micro/nano-particles TEM images: magnification × 10000, scale bar “▬” as 200 nm; **a**: porcine bone soup MNPs; **b**: UF-MNPs isolated from porcine bone soup; **c**: SEC-NPs isolated from porcine bone soup. Cryo-TEM images: magnification × 8000; **d**: porcine bone soup MNPs, scale bar “▬” as 500 nm; **e**: UF-MNPs isolated from porcine bone soup, scale bar “▬” as 500 nm; **f**: SEC-NPs isolated from porcine bone soup, scale bar “▬” as 500 nm. Green arrows: particles with diameter of 40~100 nm; Red arrows: particles with diameter of around 250 nm

It is shown in Table 1, the porcine bone soup has rich content of spherical particles (over 1 × 10^10^ particles/mL) with average hydrodynamic diameter of 229 ± 2 nm and ζ-Potential of −19.9 ± 0.4 mV. Abundant purified Micro/Nano-particles (MNPs) and nanoparticles (NPs) were obtained by ultrafiltration (molecular weight cut-off 100 kDa) and Size-Exclusion Chromatography (SEC) coupled with multi-angle laser light scattering detector (MALLS) (as shown in Fig. 1), respectively. The chromatograph of SEC separation shows the NPs from porcine bone soup has been successfully isolated by collecting the fraction with strong light scattering intensity. The smaller diameter and Particle Distribution Index (PDI) of particle fraction indicate the sufficient separation of NPs has been achieved. It is shown in Fig. 2 that particles with diameter of 40~100 nm and of around 250 nm were successfully isolated from the soup by both ultrafiltration and SEC, observed with cryo-TEM. The NPs with diameter of 40~100 nm is the major population in SEC-NPs. The molar mass of NPs fraction was further determined as 1 × 10^6^ ~ 1 × 10^8^ Da, by the SEC-HPLC on a high resolution SEC column in couple with MALLS. The molar mass of NPs proved the fraction does not contain any single molecules.

**Table 1:** Compositions and colloidal properties of micro/nano- particles derived from different food materials

	Bone soup		UF-MNPs		SEC-NPs	
	(μg/mL)	%	(μg/mL)	%	(μg/mL)	%
(a)The major compositions of micro/nano- particles derived from porcine bone soup						
Dry weight	4903 ± 29	–	3533 ± 471	–	121.8 ± 2.5	–
Protein	3476 ± 53	71	2213 ± 103	63	21.5 ± 2.5	18
Carbohydrates	73.6 ± 0.8	2	52.4 ± 0.8	2	10.4 ± 0.1	9
Lipid	687 ± 57	14	1275 ± 100	36	57.2 ± 3.0	47

(b)Physical properties of micro/nano- particles derived from porcine bone soup			
Hydrodynamic Diameter (nm)^a^	229 ± 2	248 ± 10	170 ± 1
PDI	0.482 ± 0.026	0.330 ± 0.042	0.171 ± 0.005
ζ-Potential (mV)^a^	−19.9 ± 0.4	−30.4 ± 0.7	−13.2 ± 1.7
Derived counts (kcps)^a^	24415 ± 1694	17739 ± 945	4438 ± 26
Particle number ( × 10^8^ particles/mL)	106.2 ± 7.4	77.2 ± 4.1	19.3 ± 0.6

%: percentage of protein, carbohydrates and lipid content vs. dry weight.

^a^ Measured at 37 °C by dynamic light scattering. ND: not detectable, since the particle distribution of the sample is not even enough for a sufficient optical measurements particle number.

The obtained fractions, namely UF-MNPs and SEC-NPs, were characterized to possess the average hydrodynamic diameters of 248 ± 10 nm and 170 ± 1 nm, ζ-Potential of −30.4 ± 0.7 mV and −13.2 ± 1.7 mV, respectively. The hydrodynamic size difference of UF-MNPs and SEC-NPs is rather small, wherein the difference is less than 80 nm. According to the cryo-TEM images, this size difference is caused by the removal of microparticles (diameter > 0.5 μm) in SEC isolation (as shown in Figs. 2a–c). Meanwhile, chromatographic isolation may have caused some changes in shape and compositions of the MNPs, echoing the changes in surface charge of particles, wherein the shape of SEC-NPs particles is obviously more regular than the native particles in the soup. The geometric radius (radius of gyration, *R*_g_) of SEC-NPs was determined with the multi angle laser light scattering (MALLS) as 20~50 nm (as shown in Fig. 1c), which is in good agreement with the transmission electron microscopy (TEM) measurement (Fig. 2c). Due to the hydration layer adheres to the particle surface, *R*_h_ is always greater than *R*_g_, wherein the ratios of *R*_g_/*R*_h_ suggest the shape of particles, e.g., the *R*_g_/*R*_h_ ratio of a sphere is 0.77. The *R*_g_/*R*_h_ ratio of SEC-NPs is around 0.59, therefore suggesting a near-spherical shape.

**Fig. 3:**
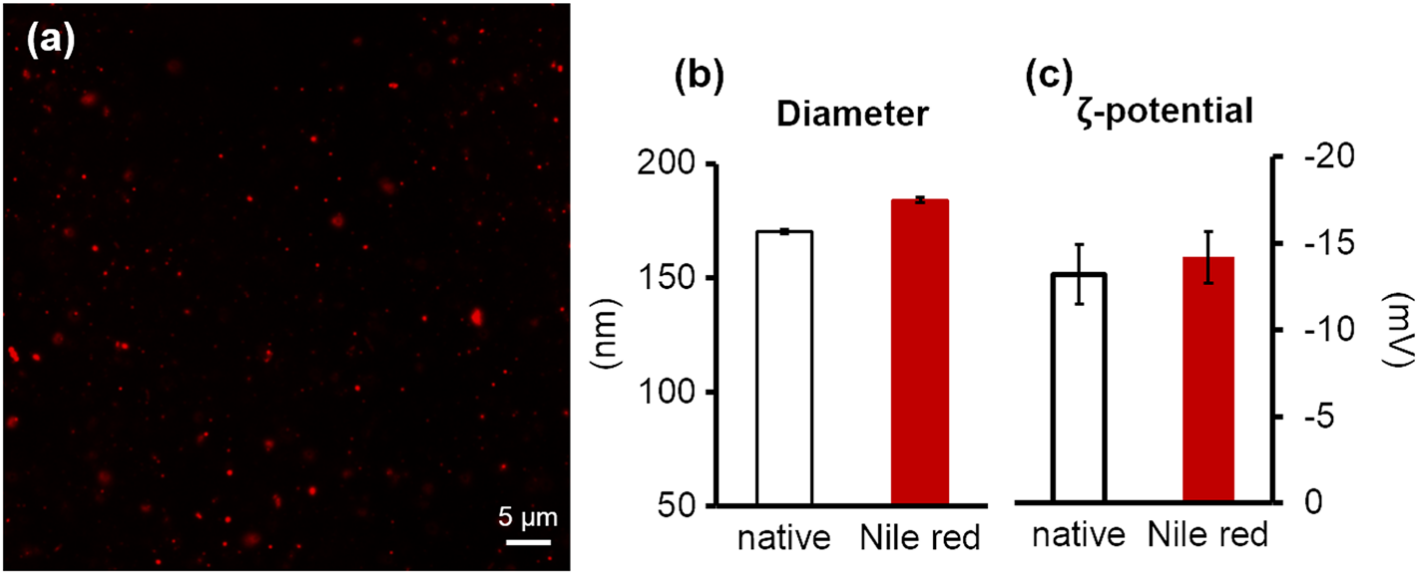
Fluorescent microscopic graph of food derived micro/nano- particles **a**: fluorescence micrograph of Nile Red stained UF-MNPs from porcine bone soup; **b** and **c**: the hydrodynamic diameter and ζ-potential of SEC-NPs, isolated from porcine bone soup, wherein no significant difference was observed before and after Nile red staining

MNPs are the major composition of soup, occupying more than 70% of the dry weight of soup. Proteins and lipids are the major composition of the UF-MNPs and SEC-NPs, in terms of weight concentration vs. weight of dry matter, containing higher content of lipid than protein in SEC-NPs and vice versa in UF-MNPs. The ζ-Potential of SEC-NPs is smaller than that of UF-MNPs, which is consistent with the lower protein content and higher lipid content in SEC-NPs since proteins are most likely the cause of negative surface charges. The particles are negatively charged and remain stable at 37 °C during the experiments including storage at 4 °C and fluorescent staining, meaning neither the secondary aggregation nor particle deconstruction has occurred. For reference, it is shown in Fig. 3 that the Nile red labeling changed neither size nor the general surface property of particles.

### Visualizing macrophage uptake of particles

**Fig. 4:**
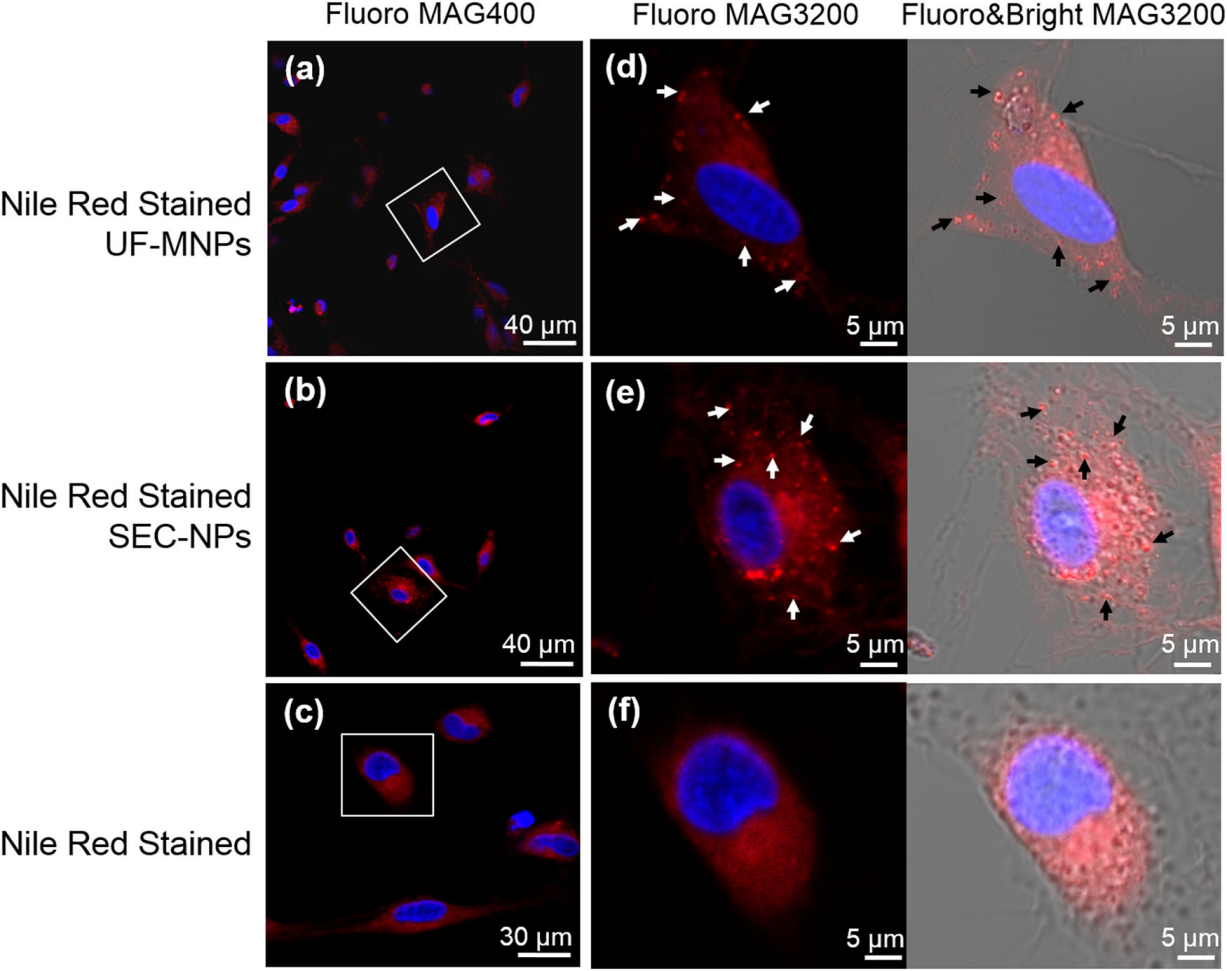
Uptake of food derived micro/nano-particles via oral mucosal macrophages. The red dots observed in cellular plasma (indicated with white or black arrows on the graphs) are Nile-red stained porcine bone soup UF-MNPs and SEC-NPs. The cell nuclei were stained in blue with a fluorescent dye Hoechst 33342. **a**–**f**: confocal fluorescence micrographs of macrophages, the particle/cell ratios were 3500/1 on oral macrophages. Cell images at different magnifications are given wherein the images of a single cell (framed with white square in diagram **a**, **b**, and **c**) are enlarged and presented as diagram **d**–**f**, scale bar = 5 μm. The red and blue fluorescent cell images are merged and presented on the left, the fluorescent and bright cell images are merged and presented on the right

Murine oral macrophages were freshly collected from oral cavity. It is shown in Fig. 2, once being incubated with Nile red stained MNPs and NPs, cellular plasma and membrane started to display fluorescent red color and became fully stained after 20 min. As any free dye of Nile Red has been thoroughly removed before the incubation, the red color displayed must be the results of the uptake of Nile Red labeled MNPs and NPs by macrophages. This is further support by the fact that the micro-/nano-size red fluorescent particulates were observed only in MNPs and NPs engulfed cells, not in cells directly stained with Nile Red. A portion of internalized particles may remain intact or form aggregates, being visualized as micro-particulates or nano-particulates in cytosolic compartments. Neither MNPs nor NPs were seen in cell nuclei (as shown in Fig. 4a–b, d–e). No visible deconstruction or acute toxic response was observed in these cells.

### Influence on the macrophage cell membrane potential (*V*_mem_)

**Fig. 5:**
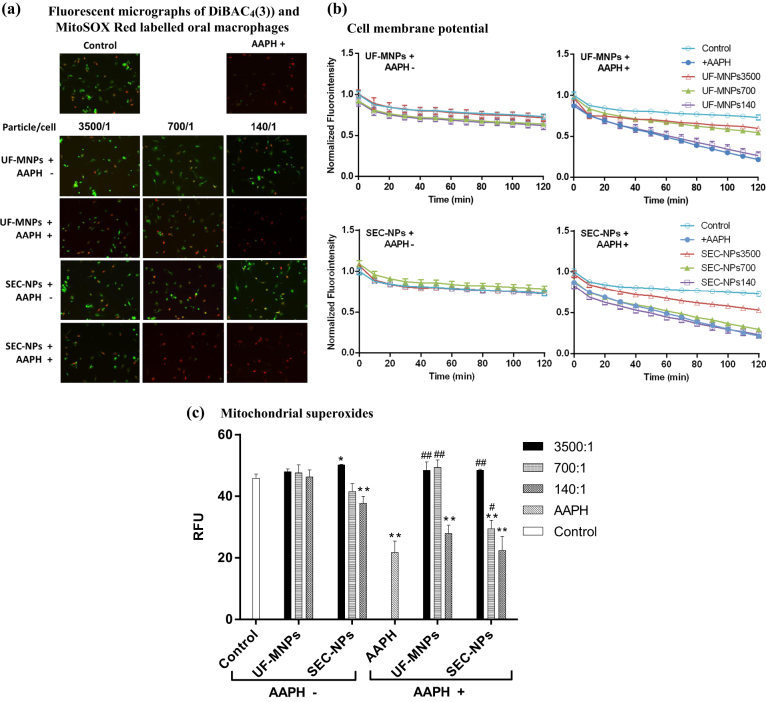
Effects of food derived micro/nano-particles on cell plasma membrane potential (indicated with green fluorescence of DiBAC_4_(3)) and mitochondrial superoxide (indicated with red fluorescence of MitoSOX) of murine oral mucosal macrophages. Cells were loaded with 3 μM MitoSOX Red for 10 min and then 2.5 μM DiBAC_4_(3) for 15 min at 37 °C. For AAPH treated cells (6.4 μM, incubated for 120 min), they were loaded with fluorescence dyes prior to the addition of AAPH and micro/nano-particles. **a**: Fluorescent micrographs (red & green fluorescence, taken by a Leica DMI3000 B Inverted Microscope, excitation at 515~560 nm and 450~490 nm, respectively) of loaded cells, merged with software LAS (version 4.2.0) wherein UF-MNPs/cell ratio = 3500/1,700/1 and 140/1; SEC-NPs/cell ratio = 3500/1,700/1 and 140/1. **b**: *V*_mem_-dependent fluorescence traces (1 data-point/10 min for 120 min, excitation/emission at 493/516 nm for DiBAC_4_(3)) of loaded macrophages in a 96-well microplate were measured simultaneously by a fluorometric plate reader (Flexstation3, Molecular Devices, USA). **c**: the diagram shows the impacts of UF-MNPs, SEC-NPs and AAPH on the mitochondrial superoxide level of macrophages, indicated by MitoSOX Red fluorescence (excitation/emission at 510/580 nm). *: *P* < 0.05 v.s. Control, **: *P* < 0.01 v.s. Control; #: *P* < 0.05 v.s. AAPH, ##: *P* < 0.01 v.s. AAPH. The oral macrophages partially presented green-yellowish color due to the overlap between two fluorescent probes in cell compartments or cell groups. Among normal macrophages, cells with higher level of mitochondrial superoxide (brighter red fluorescence) tend to have hyperpolarized membranes (weaker green fluorescence) which carry more intensively the negative charge. AAPH induced peroxyl radicals eliminated the DiBAC_4_(3) fluorescence as well as decreased MitoSOX Red fluorescence, indicating the hyperpolarization of cell membrane and down-regulated mitochondrial oxygen respiratory

It is shown in Fig. 5, the engulfment of either UF-MNPs or SEC-NPs did not affect the green fluorescence emitted by the membrane potential sensitive dye DiBAC_4_(3) in oral macrophages at all assayed particle concentrations, indicating the MNPs and NPs do not influence the *V*_mem_ of macrophages, even at a very high particle/cell ratio of 3500/1.

At the presence of AAPH, DiBAC_4_(3) fluorescence in oral macrophages was eliminated (Fig. 5a), reporting the rising *V*_mem_ and hyperpolarization of cellular plasma membrane caused by AAPH-induced peroxyl radicals. Membrane hyperpolarization is known to be paralleled with the activation of macrophage functions, i.e., NO production.^22,23^

In contrast, both UF-MNPs and SEC-NPs completely or partially restored the *V*_mem_ in AAPH insulted cells in a dose-dependent manner (Fig. 5b), counteracted the membrane hyperpolarization caused by the extracellular peroxyl radicals. Judging by the higher fluorescence intensity, UF-MNPs have a more prominent effect than SEC-NPs. The MNPs and NPs sufficiently inhibited ROS-induced hyperpolarization at the particle/cell ratios of 3500/1 and 700/1. Therefore, porcine bone soup MNPs and NPs did not affect cytosolic membrane of normal macrophages, but could protect cytosolic membrane from AAPH-induced oxidative damage.

### Influence on macrophage mitochondrial superoxide content

Mitochondrial ROS plays a vital role in the signaling, phagocytosis, and immunology response of macrophages to intracellular or extracellular stimuli.

It is shown in Fig. 5c, engulfment of either UF-MNPs or SEC-NPs did not affect the fluorescence of ROS sensitive dye MitoSOX Red (also named Mito-HE) in oral macrophages, whereas uptake of SEC-NPs at the lowest concentration slightly reduced the Mito-HE fluorescence (particle/cell = 140/1). It implies bone soup MNPs and NPs exert minor influence on mitochondrial ROS.

In contrast, at the presence of AAPH, Mito-HE fluorescence was largely reduced, reporting significant suppression on mitochondrial oxygen respiration and therefore ROS generation. In contrast, UF-MNPs and SEC-NPs restored the Mito-HE fluorescence, counteracted the AAPH-induced suppression on mitochondrial ROS, particularly at the highest concentration tested, i.e., particle/cell ratio 3500/1. At the lowest dose, which was 1/25 of the highest dose, neither UF-MNPs nor SEC-NPs affected the ROS generation in mitochondria of cells challenged by AAPH. Similar to the effects on *V*_mem_, UF-MNPs induced stronger red fluorescence than SEC-NPs at medium concentration (700/1), indicating the higher capacity of UF-MNPs on regulating mitochondrial ROS.

It implies internalization of bone soup MNPs and NPs can help maintain the mitochondrial oxygen respiration of macrophages, despite of peroxyl radicals induced oxidative damages.

## Discussion

Using size-exclusive chromatography, the lipid/protein-containing porcine bone soup NPs are satisfactorily isolated and exhibit typical nano-scale size, high molecular mass of polymers (>10^6^ Da), narrow particle distribution, unified and identical surface appearance, indicating a fine separation of nanoparticles from the soup has been achieved. The same approach has been applied on the separation of artificial NPs^24^ and self-assembled NPs from herbal materials,^7^ while this work is extending this approach to a complex multiphase dispersion system of food. The details of this chromatographic isolation will be documented separately, to discuss the possible application in many other food systems.

As describe above, the isolated UF-MNPs and SEC-NPs inherit some general profiles of micro-/nano-aggregates in porcine bone soup, in terms of chemical compositions, morphological characteristics, and surface properties. However, the significant differences were also found in the protein content, lipid content, and ζ-potential of isolated particulates, indicating either the composition variation between the particles in micro-meter and nano-meter ranges, or the chemical and structural alteration caused by the rather ‘harsh’ separation process. The membrane filtration (ultrafiltration) and gel-filtration (SEC) involve deconstructing force, i.e., applied pressure, osmotic pressure, frictional force, and shear force of the flow fluid around particles in tortuous channels of membrane and chromatographic resin, which may possibly modify the composition or structure of colloidal particles.^25,26^ The possible influence of these separation-induced changes on the function and bioactivities of MNPs has to be put into consideration when evaluating the particle-cell interaction of isolated particles and the native colloidal particles of porcine bone soup. Meanwhile, the bone soup, with its full composition including native MNPs, has demonstrated significant intracellular ROS scavenging activities on four different kinds of cells. ^21^ It is in the similar way that UF-MNPs and SEC-NPs have protected macrophages from peroxyl radical oxidation. The similarity found in the cellular activities of native particle and separated particles implies the UF-MNPs and SEC-NPs may have reserved some key characteristics of the original soup MNPs.

Furthermore, despite the above differences, these MNPs and NPs are stable enough to survive the staining process of fluorescent dyes, and even stay intact after being engulfed by macrophage. In a way, the successful survival of these micro/nano-structures through different challenging processes, e.g., separation and staining, reconfirms the necessity of studying the MNPs in food on top of their constituent compositions.

Mucosal macrophages are selectively responding to particles, depending on the physiochemical properties of particles, whereas discrepant results have been reported using different particle building materials and cell types. For nano-vesicles of liposomes, negatively charged vesicles are more readily taken up by macrophages than positively charged or neutral vesicles,^27^ while the liposome size of unilamellar vesicles (diameter 25–160 nm) is reversely correlated to their uptake by macrophages.^28,29^ In contrast, the cationic or less negative poly (lactic-co-glycolic acid) (PLGA) nanoparticles are more tend to be internalized by “macrophage-like” cells than the unmodified PLGA NPs and microparticles.^30,31^ In another study carried out with chitosan nanoparticles (diameter 150–500 nm, ζ-potential −40 mV to + 35 mV), NPs with high surface charge and large particle size were more efficiently phagocytized by murine macrophages.^32^

In the current study, factors determining the engulfment of NPs by macrophages are yet to be elucidated, the more negatively charged surface (ζ-potential −30.4 mV vs. −13.2 mV) and higher content of protein in UF-MNPs may result in the higher efficiency of macrophage uptake, and the stronger inhibition of UF-MNPs on AAPH-induced membrane hyperpolarization and mitochondrial dysfunction.

On the other hand, various types of MNPs have been extensively studied for the potential application in drug and bioactives delivery. However, it has been recognized that the artificially prepared nano-assemblies, either organic or non-organic, are often stimuli to mucosal cells, e.g., macrophages. For instance, TiO_2_-MNPs seduced superoxide anion in alveolar macrophages.^33^ Cationic polystyrene NPs (amine-modified) disrupts the membrane potential and induces membrane depolarization on epithelial cells, whereas anionic polystyrene NPs (carboxylate-modified) neither cause depolarization nor alter oxygen respiratory in mitochondria.^16^ Biodegradable MNPs, e.g., PLGA microparticles (5–7 μm), can attach to the outer layer of plasma membrane of J774.A1 cells and seduce potent inflammatory responses.^34^

Comparing with the above mentioned toxic effects of MNPs and NPs on the engulfing cells, internalization of bone soup UF-MNPs and SEC-NPs neither disturb the cell plasma membrane nor affect the intracellular superoxide in the mitochondria. In another word, exposure to bone soup micro-particles and/or nano-particles does not irritate macrophages, which is consistent with the above studies conducted with anionic polymeric NPs, and is consistent with the particle dosages employed in this study are far lower than the non-cytotoxic concentration of bone soup. The anionic surface of bone soup particles may make them compatible to macrophages. Furthermore, it is intriguing to see the internalized bone soup particles remain intact in cellular plasma during 3–4 h of observation, without being degraded by process like phagocytosis or triggering the cell deformation. It appears the bone soup particles may possibly be transported across the mucosa as an intact unit.

Macrophages are in the frontline of body immune response with an excessive tolerance to endogenous or environmental ROS. The azo compound AAPH was used in this study to generate peroxyl radicals, simulating the ROS challenges macrophages may encounter in the microenvironment of alimentary tract during food ingestion. AAPH can induce lipid peroxidation on cell membrane of macrophages and subsequently cause the formation of rigid domains like ‘lipid rafts’ or gel phase domains^35,36^ and disturbed membrane potential.^37^ Several independent studies suggest that rigid lipid domains and changes in membrane fluidity are correlated to motility, activation, and phagocytosis of macrophages.^35,38^ Studies have also demonstrated that cell plasma membrane hyperpolarization increases the Ca^2+^ influx through voltage-independent ion channels and is correlated to less active mitochondria, in quiescent (non-proliferating) cells for instance.^39,40^ The influences of AAPH on oral macrophage described here is consistent with the previous reports. As it may imply, UF-MNPs and SEC-NPs restore *V*_mem_ to the normal resting level and therefore protected macrophages’ cellular membrane and mitochondria from AAPH-induced peroxyl radical oxidation, indicating a pacifying effect upon the irritated macrophages. Although its mechanism remains unknown, it is clear that the bone soup particles may help to maintain the homeostasis of macrophage, echoing the general belief in the immunity modulating power of bone soup perceived in the folk practice.

Although the in vivo process of food derived MNPs ingestion ought to be far more complicated, it is still reasonable to speculate whether the bone soup MNPs and NPs are ingested by the enterocytes and lymphatic vessel and transported to blood, as do the chylomicrons. Instead, it is rather rational to propose an alternative mechanism for the absorption, transportation and utilization of micro-/nano-particles in food, starting from the direct uptake by mucosal cells including macrophages.

However, the cellular impacts of bone soup particles were observed in a time scale of minutes, while the ingestion of liquidized food in the mouth may often be a matter of seconds. It remains unclear whether the food MNPs could be quickly engulfed in the mouth during eating and drinking, thereafter be sufficient to cause any relevant physiological outcomes. Further studies are warranted to illuminate whether the mucosal macrophages engulf food MNPs in vivo, whether it is energy dependent or independent, and whether it acquires the participation of enterocytes, e.g., microfold cells at Peyer’s Patch. Paralleling to their chemically synthetic cousin, NPs from the dietary soup are safe, stable, and easy to prepare, providing a new perspective and prototype for developing new nano-vehicles for drug and nutrients.

## Methods

### Preparation of porcine bone soup

The fresh porcine femur bones from the Landrace pigs (*Sus scrofa*) were purchase from local market, chopped into fragments of 3 × 3 cm. Blood residues were removed with 1% sodium citrate solution by soaking for 30 min and subsequently washed with still water for 3 times. The bone (1000 g) was cooked with deionized water at 1:3(W/V) in a 5-liter beaker sitting in boiling water bath (100 °C under normal air pressure) for 3 h, covered with a lid and heated by an electromagnetic oven. The water level in the water batch was kept slightly higher than the water level in the beaker by adding a small amount of boiling water every hour. The real-time soup temperature was monitored with a thermometer soaking in the beaker. The soup temperature remained stable at 98 ± 1 °C. The soup was then collected by filtering through a gauze to remove the residues of meat and bone tissue, stored at −20 °C. The overall light scattering intensity, average hydrodynamic diameter, dry weight, protein, carbohydrates, and lipid content of the soup from individual cooking were determined for monitoring the reproductivity of soup cooking. The complete cooking process was repeated for 3 times to calculate the mean values and standard deviation. The sterilized emulsion of bone soup remained stable during a 5-day stability test at 37 °C.

### Isolation of bone soup UF-MNPs and SEC-NPs

UF-MNPs: 5 mL freshly prepared bone soup was moved into an ultrafiltration tube (MW cut-off 100 kDa; catalog number: UFC910008; Millipore, USA) with a sterile transfer pipet and spun at 5000 g (Model CF16RX, Hitachi Koki Co., Ltd., Japan), 40 °C for 20 min. Remove the supernatant, wash with Hank’s balanced salt solution (HBSS, Gibco, USA, catalog number: 14025076) for 3 times, re-suspend the particles (UF-MNPs) with HBSS and measure the particle number (light scattering intensity). Bring particle concentration to the appropriate number.

SEC-NPs: nanoparticles were separated from bone soup by chromatographic method.^7^ Briefly, bone soup was centrifuged at 400 g for 10 min. The supernatant (2 mL) bone soup was applied to a pre-equilibrated size-exclusive chromatographic column (Sephacryl S-1000 SF from GE Health, USA, 1.0 × 100 cm, pH7.4, 0.02 M) equipped with a FPLC system (BioLogic DuoFlow 10, Bio-Rad, USA) at 40 °C (column with water bath jacket), flow rate 0.5 mL/min, UV monitor 280 nm coupling with Zetasizer Nano-ZS (Malvern Instruments Ltd., UK) with a flow-cell (MODEL:ZEN0023, Malvern Instruments Ltd., UK) installed. The fraction of high-light scattering was collected (BioFrac Fraction Collector, Biorad, USA) and named as SEC-NPs.

The further characterization of particles was performed as early as possible after the separation. The unused particle samples were either stored at 4 °C for short term experiments and discarded after 2 days, or stored at −20 °C in small proportions (2 ml each) for longer term of use and discarded after 2 months. The repeatedly freeze-thaw was avoided by using a new portion of sample each time. The hydrodynamic diameter, ζ-potential and light scattering intensity of particles were performed for every particle sample, i.e., after the thawing or before the incubation with cells, to monitoring the possible changes in the characteristics of MNPs.

### Molar mass characterization of SEC-NPs

The fraction SEC-NPs was applied to a TSKgel G6000PWxl column (0.78 × 300 cm, Tosoh Bioscience, Japan) with a HPLC system (Quest 10 Plus, Bio-Rad, USA), eluted with the phosphate buffer (0.1 M, pH7.4) at a flow rate of 0.80 mL/min. The eluates were continuously monitored with a UV detector and a multi-angle laser light scattering detector (MALLS, DAWN HELEOS II, Wyatt Technology, CA, USA) to obtain the absorbance at 280 nm and light scattering intensity at 658 nm. The exclusion of single molecules in SEC-NPs was confirmed by calculating the geometric radius distribution of chromatographic peak of SEC-NPs.

### Characterization of MNPs and NPs

Particles are characterized with Zetasizer Nano-ZS (Malvern Instruments Ltd., UK) at 25 °C for their hydrodynamic diameter, ζ-potential, PDI, and quantified with NanoSight NS300 system (Malvern Instruments Ltd., UK) at 25 °C. Then particles are transferred to the 300 mesh copper grid and stained with 5% uranyl acetate. The copper grid was observed on a TEM (TEM, Joel JEM-1230, Japan) at 80 kV.

For Cryo-TEM observation, the frozen hydrated samples of bone soup MNPs were prepared by applying the particles dispersion to a piece of holey carbon film (2 μm hole diameter) affixed EM specimen grid. The grid was blotted with filter paper to remove excess fluid. For preventing the formation of ice crystals, the specimen grid was rapidly plunged into liquid ethane at liquid nitrogen temperature and then transferred to the TEM by a precooled cryo-stage within a cryo transfer holder. The bone soup MNPs and UF-MNPs were observed with a FEI TecnaiG2 Spirit (Thermo Fisher Scientific, USA) at 120 kV. The SEC-NPs were observed with a FEI Talos F200C (Thermo Fisher Scientific, USA) at 200 kV.

Protein content was determined by Bicinchoninic acid (BCA) assay (Kit: 20170112. Nanjing Jiancheng Bioengineering Institute, China) with bovine serum albumin as standard sample.^41^ Polysaccharides content was determined by anthrone–sulfuric acid assay^42^ with glucose as standard sample. Lipid content: triglyceride content was determined with GPO-PAP assay (Kit: 20170112, Nanjing Jiancheng Bioengineering Institute, China).^43^

### Preparation of oral macrophages

Rats (Wistar, male, age 8–12 weeks, SPF grade) were euthanize by rapid cervical dislocation. The corpses were sterilized by soaking in 70% ethanol for 5 min. cut off the cheeks and soft palate in oral cavity wall, wash with cold PBS (4 °C, penicillin 100 U/Ml, streptomycin 100 μg/mL) for 4 times. Remove facia and blood. The tissue is cut to tiny pieces of 1 mm^3^ with sterile scissor or surgical knife, and then placed in warm digestion medium (DMEM without serum, 0.075% Type I collagenase, 0.075% Type II collagenase) at 37 °C for 2 h, shaking every 5 min. The cell suspension was collected by filtering through a 100 μm Nylon cell strainer (Falcon, catalog number: 352360) to remove the indigested tissue. Spin and wash the cells twice with DMEM medium, each time 5 min at 400 g. Macrophages were further purified on a density gradient by applying cell suspension on top of Histopaque 1077 (Sigma–Aldrich, catalog number: 10771) at 1:1 (V/V) and spinning at 20 °C, 400 g for 20 min. Collect the bulky layer of cells at the interface of two solutions and wash with DMEM medium. Re-suspend the cells in warm DMEM medium (15%FBS, penicillin 100 U/Ml, streptomycin 100 μg/mL) and bring cell concentration to 1 × 10^6^ cells/mL. Seed into T-75 cell culture flasks (Coring, USA, catalog number: 430641), place into incubator (37 °C, 5%CO_2_) for 2 h to allow macrophages to adhere and then remove the suspending cells. Refill the bottle with full DMEM medium and incubate overnight, then digested with 0.25% trypsin-EDTA (Gibco, USA, catalog number: 25200-056). Bring cell concentration to appropriate concentration and seed into 24-well or 96-well plates.

The animal experiment mentioned above was inspected and approved by the Animal Care & Welfare Committee of Zhejiang Academy of Medical Sciences, China (No. 2016R10031).

### Visualize macrophage uptake of particles

Nile red labeling: add Nile red to the particles dispersion at 1 μg/mL, 40 °C for 1 h. The unbound dye was removed by repeatedly wash the retained particles with HBSS in an ultrafiltration tube (MW cut-off 100 kDa; catalog number: UFC910008; Millipore, USA) till no fluorescence is observed in the filtrate. The retained particles are re-suspended in HBSS. Macrophages are seeded in 24-well plates with three duplicates (2 × 10^4^ cells/mL, 0.6 mL per well, Cellvis, USA, catalog number: P24-1.5H-N) and place into an incubator (37 °C, 5%CO_2_) overnight. Remove the culture medium, then add Nile red labeled particles. The particle/cell ratios are 14,000/1, which is 10 times lower than the non-cytotoxic concentration of bone soup determined by MTT assay.^23^ The cell nuclei were stained in blue with a fluorescent dye Hoechst 33342. Fluorescent microscopic observation is performed with a Zeiss LSM 780 Laser Scanning Confocal Microscope (Carl Zeiss SAS, Germany; Ex = 549 nm, Em = 628 nm for Nile red, Ex = 346 nm, Em = 460 nm for Hoechst 33342). The red and blue fluorescent images are merged with Zen software (Zeiss). The whole process was repeated twice to confirm the reproducibility.

### Cell membrane potential and mitochondrial superoxide determination

Macrophages are seeded in 96-well plates (2 × 10^4^ cells/well, black&flat-bottom wells, Coring, USA, catalog number: 3603), wash with HBSS, and stain with Mito-SOX Red (200 μL, 3 μM in HBSS) for 10 min at 37 °C, then remove working solution, wash with HBSS for 2 times. Bis-(1,3-dibutylbarbituric acid) trimethine oxonol (DiBAC_4_(3), Sigma–Aldrich, USA, catalog number: D8189) was added to each well (100 μL, 5 μM) and place into an incubator (37 °C, 5%CO_2_) for 15 min. Bone soup samples (50 μL/well) are added at different particle/cell ratios, using HBSS as control. The particle/cell ratios are 3500/1, 700/1, 140/1. 2,2′-azobis (2-amidinopropane) dihydrochloride (AAPH, Sigma–Aldrich, USA, catalog number:440914, 25.6 μM in HBSS) is added (50 μL/well, final concentration 6.4 μM). The fluorometric measurements are conducted on three duplicates by FlexStation 3 Plate Reader (Molecular Devices, Sunnyvale, CA, USA) every 10 min for 120 min at 37 °C for cells stained with Mito-SOX Red and DiBAC_4_(3), at Ex = 510 nm, Em = 580 nm and Ex = 493 nm, Em = 516 nm, respectively. The fluorescent micrographs are taken right after the fluorometric measurement (Leica, DMI3000 B Inverted Microscope, Ex = 515–560 nm and Em = 590 nm for MitoSOX Red, Ex = 450~490 nm and Em = 516 nm for DiBAC_4_(3)). The whole process was repeated twice to check the reproducibility.

### Statistics

The significant levels were examined with Student’s *t*-test (two-sided) and ranked as *P* < 0.05 or *P* < 0.01, and error bars as s.d.

### Data availability

The authors declare that the data supporting the findings of this study are available within the article.^44,45^
